# Supplementary material for: FZD10 regulates cell proliferation and mediates Wnt1 induced neurogenesis in the developing spinal cord
Source: PLoS One. 2020 Jun 12;15(6):e0219721. doi: 10.1371/journal.pone.0219721 (PMC7292682; doi:10.1371/journal.pone.0219721)
Supplement: S1 Fig — (A-F) Cryosections of embryos 48 hours after electroporation into the neural tube (A, D, C, F) RFP expression indicates the electroporated side. (B, C) FZD10 expression was not affected after electroporation of scrambled shRNA. (E, F) FZD10 expression was reduced after electroporation of FZD10 shRNA vectors. RFP was detected by immunostains on cryosections after whole mount in situ hybridization. (DOCX) [file pone.0219721.s001.docx]

**
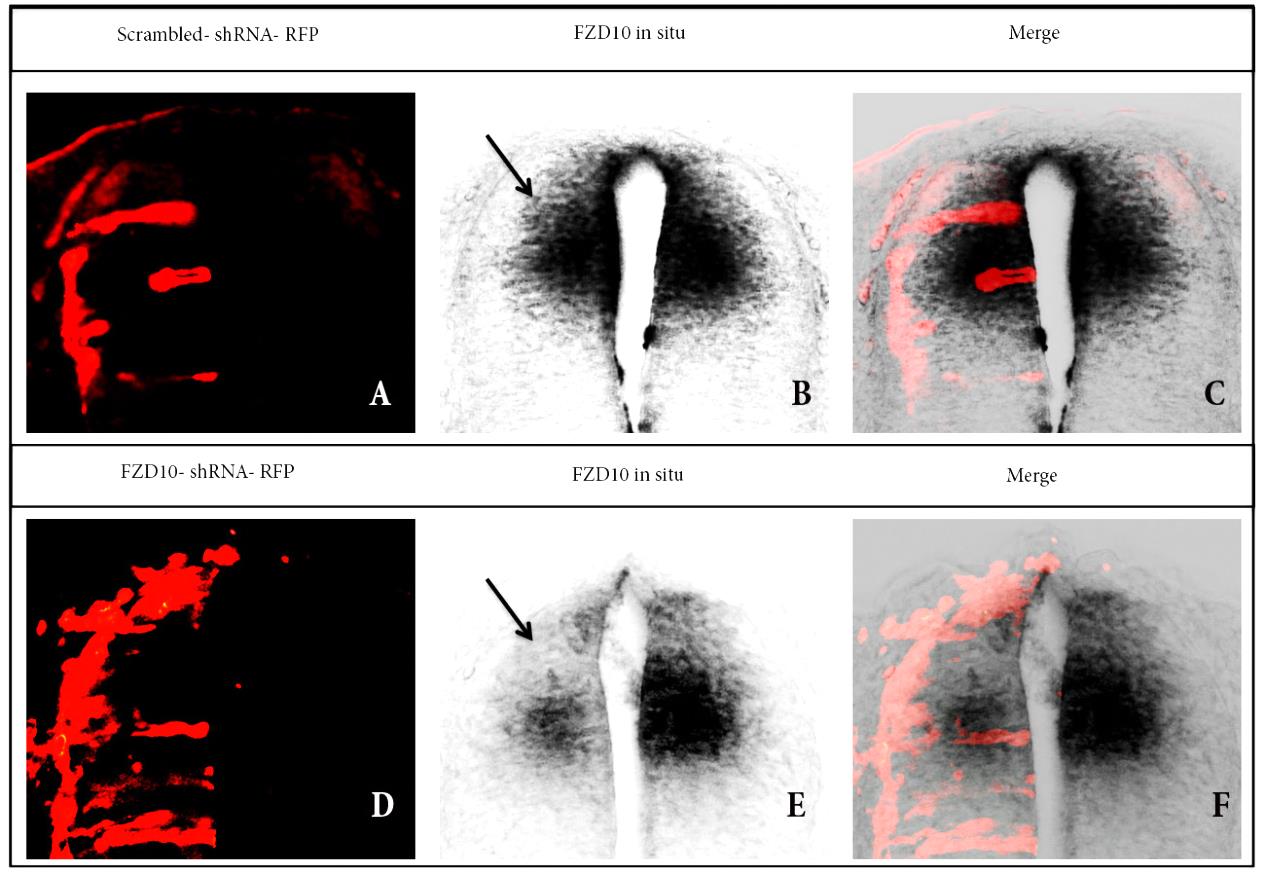
**

S1 Fig: FZD10 expression was knocked down by FZD10 shRNA vectors. (A-F) Cryosections of embryos 48 hours after electroporation into the neural tube (A, D, C, F) RFP expression indicates the electroporated side. (B, C) FZD10 expression was not affected after electroporation of scrambled shRNA. (E, F) FZD10 expression was reduced after electroporation of FZD10 shRNA vectors. RFP was detected by immunostains on cryosections after whole mount in situ hybridization.
