# Supplementary material for: FZD10 regulates cell proliferation and mediates Wnt1 induced neurogenesis in the developing spinal cord
Source: PLoS One. 2020 Jun 12;15(6):e0219721. doi: 10.1371/journal.pone.0219721 (PMC7292682; doi:10.1371/journal.pone.0219721)
Supplement: S2 Fig — Scrambled shRNA or FZD10 shRNA vectors (as indicated) were electroporated into neural tubes at stage 11–12 and effects were analysed immunohistochemistry. (A-C) The expression domain of Pax7 was identical on both sides of the spinal cord. (D-F) The ventral extend of Pax7 expression was reduced on the eletroporated side. (G-I) Tuj-1 expression was repressed on the eletroporated side of the spinal cord, suggesting that FZD10 is required for the differentiation of interneurons. The number of embryos was 3 for each marker and condition, >10 sections were analysed for each marker. (DOCX) [file pone.0219721.s002.docx]

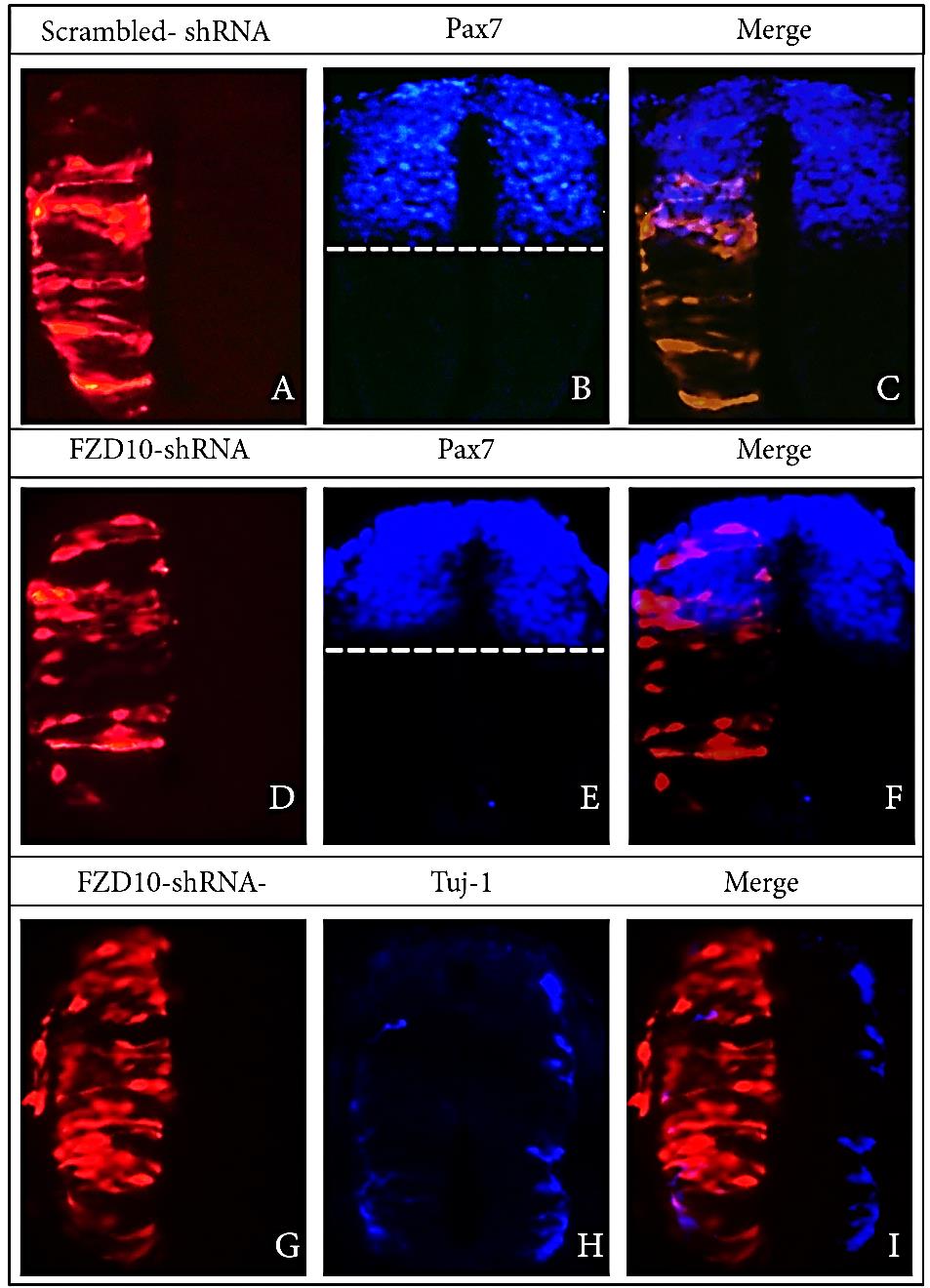


S2 Fig: FZD10 knockdown affects neural markers in spinal cord within 24 hours. Scrambled shRNA or FZD10 shRNA vectors (as indicated) were electroporated into neural tubes at stage 11-12 and effects were analysed immunohistochemistry. (A-C) The expression domain of Pax7 was identical on both sides of the spinal cord. (D-F) The ventral extend of Pax7 expression was reduced on the eletroporated side. (G-I) Tuj-1 expression was repressed on the eletroporated side of the spinal cord, suggesting that FZD10 is required for the differentiation of interneurons. The number of embryos was 3 for each marker and condition, >10 sections were analysed for each marker.
