# Supplementary material for: FZD10 regulates cell proliferation and mediates Wnt1 induced neurogenesis in the developing spinal cord
Source: PLoS One. 2020 Jun 12;15(6):e0219721. doi: 10.1371/journal.pone.0219721 (PMC7292682; doi:10.1371/journal.pone.0219721)
Supplement: S4 Fig — GFP expression in the transfected sides is shown in green. (A, B) Expression of dorsal markers, Pax7 and Pax6 is expanded ventrally on the experimental side. (C) Expression of Nkx2.2 is repressed and shifted ventrally. (DOCX) [file pone.0219721.s004.docx]

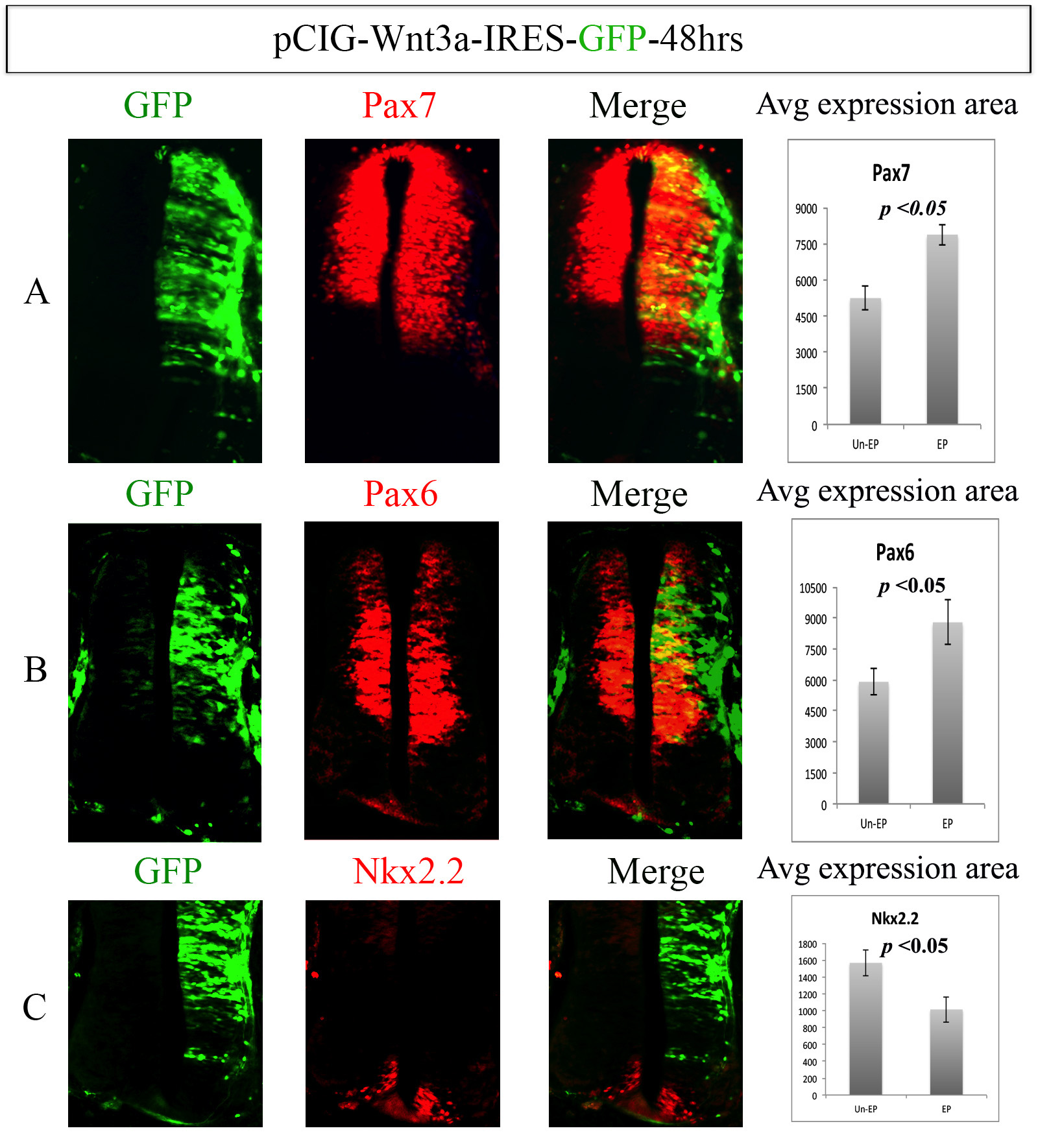


**S4 Fig:**  **Analysis of expression patterns of neural markers after transfection of Wnt3a into the spinal cord.** GFP expression in the transfected sides is shown in green. (A, B) Expression of dorsal markers, Pax7 and Pax6 is expanded ventrally on the experimental side. (C) Expression of Nkx2.2 is repressed and shifted ventrally.
