# Supplementary material for: FZD10 regulates cell proliferation and mediates Wnt1 induced neurogenesis in the developing spinal cord
Source: PLoS One. 2020 Jun 12;15(6):e0219721. doi: 10.1371/journal.pone.0219721 (PMC7292682; doi:10.1371/journal.pone.0219721)
Supplement: S5 Fig — Whole mount in situ and sections were used to establish expression profiles of Wnt1, Wnt3a and FZD10 in HH14 and HH20 chick embryos. (A-F) Dorsal views of whole mount embryos shows expression patterns of Wnt1, Wnt3a and FZD10 as indicated. (a-f) Corresponding transverse sections of chick HH14 and HH20 shows Wnt1, Wnt3a and FZD10 expression in dorsal regions of the spinal cord. (DOCX) [file pone.0219721.s005.docx]

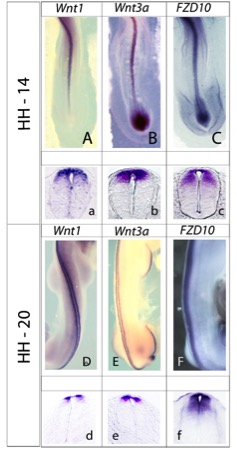


**S5 Fig:** **FZD10 expression overlaps with Wnt1 and Wnt3a in the spinal cord.**

Whole mount in situ and sections were used to establish expression profiles of Wnt1, Wnt3a and FZD10 in HH14 and HH20 chick embryos. (A-F) Dorsal views of whole mount embryos shows expression patterns of Wnt1, Wnt3a and FZD10 as indicated. (a-f) Corresponding transverse sections of chick HH14 and HH20 shows Wnt1, Wnt3a and FZD10 expression in dorsal regions of the spinal cord.
