# Supplementary material for: FZD10 regulates cell proliferation and mediates Wnt1 induced neurogenesis in the developing spinal cord
Source: PLoS One. 2020 Jun 12;15(6):e0219721. doi: 10.1371/journal.pone.0219721 (PMC7292682; doi:10.1371/journal.pone.0219721)
Supplement: S6 Fig — (A, D, G) GFP expression on the transfected side of the spinal cord, indicating that FZD10 is ectopically expressed. (B, C) The Pax7 and (E, F) the Pax6 expression domains are shifted dorsally on the electroporated sides. (H, I) The Nkx2.2 expression domain is dorsally expanded on the electroporated side. (DOCX) [file pone.0219721.s006.docx]

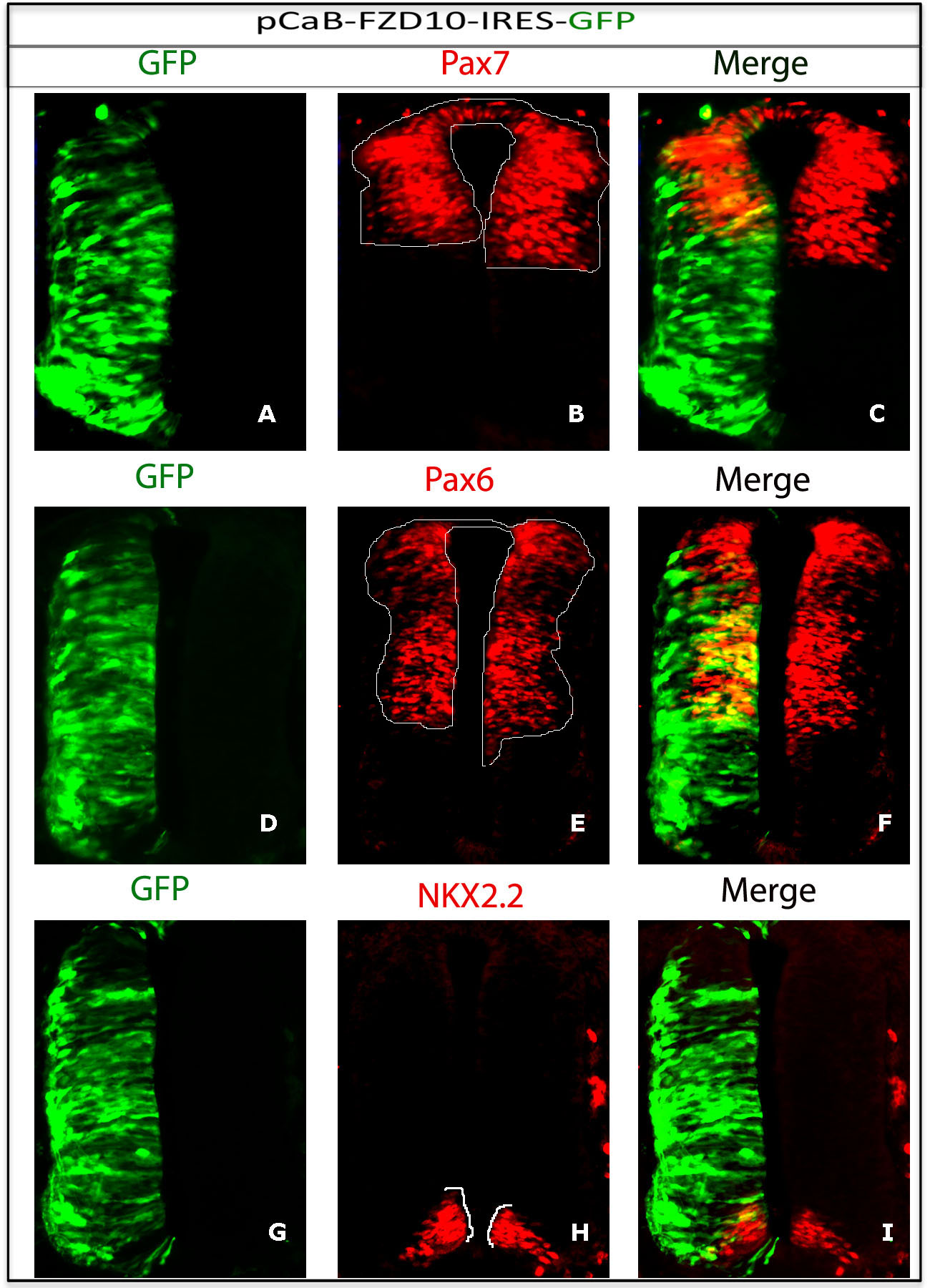


**S6 Fig:** **FZD10 overexpression affects neural progenitor pattering along the in D-V axis of the spinal cord.** (A, D, G) GFP expression on the transfected side of the spinal cord, indicating that FZD10 is ectopically expressed. (B, C) The Pax7 and (E, F) the Pax6 expression domains are shifted dorsally on the electroporated sides. (H, I) The Nkx2.2 expression domain is dorsally expanded on the electroporated side.
