# Supplementary material for: FZD10 regulates cell proliferation and mediates Wnt1 induced neurogenesis in the developing spinal cord
Source: PLoS One. 2020 Jun 12;15(6):e0219721. doi: 10.1371/journal.pone.0219721 (PMC7292682; doi:10.1371/journal.pone.0219721)
Supplement: S7 Fig — (A) Pax7 expression 48 hours after Wnt1 electroporation. (B) Pax7 expression after co-transfection of Wnt1 with FZD10 and Lrp6. The white bracket in A, B indicates the ventral expansion that was measured. (C) The average length of Pax7 expansion in both experiments; ventral expansion of Pax7 was enhanced 2-fold after introducing both FZD10 and Lrp6 together with Wnt1 into the spinal cord. (DOCX) [file pone.0219721.s007.docx]

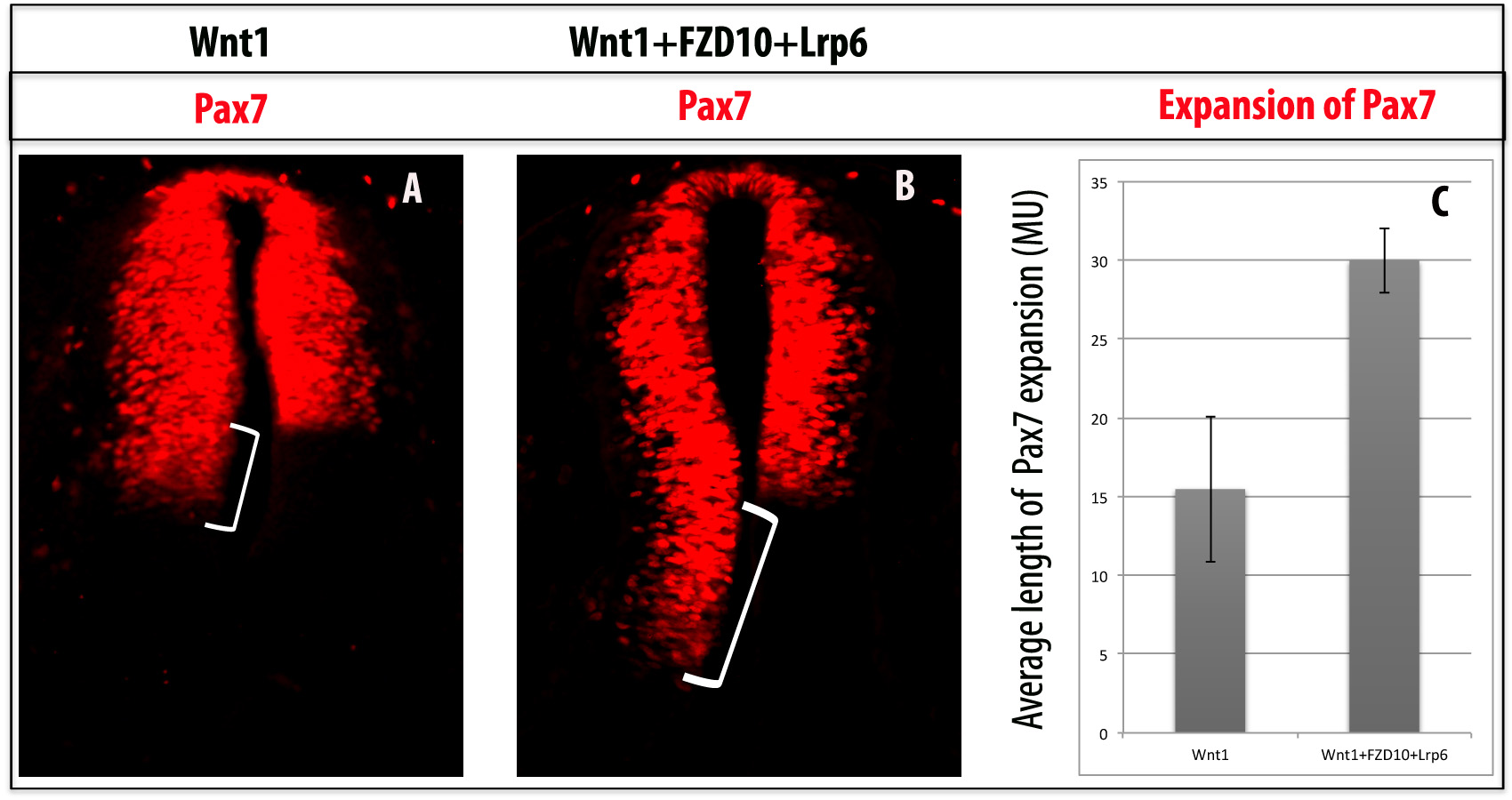


S7 Fig: Ventral expansion of Pax7 is enhanced after transfection Wnt1, FZD10 and Lrp6 compared to Wnt1 alone. (A) Pax7 expression 48 hours after Wnt1 electroporation. (B) Pax7 expression after co-transfection of Wnt1 with FZD10 and Lrp6. The white bracket in A, B indicates the ventral expansion that was measured. (C) The average length of Pax7 expansion in both experiments; ventral expansion of Pax7 was enhanced 2-fold after introducing both FZD10 and Lrp6 together with Wnt1 into the spinal cord.
