# Supplementary material for: FZD10 regulates cell proliferation and mediates Wnt1 induced neurogenesis in the developing spinal cord
Source: PLoS One. 2020 Jun 12;15(6):e0219721. doi: 10.1371/journal.pone.0219721 (PMC7292682; doi:10.1371/journal.pone.0219721)
Supplement: S1 Chart — (DOCX) [file pone.0219721.s008.docx]

**S1 Chart:** **Summary of rescue experiments that shows numbers of embryos and their phenotypes for each condition.**
